# The Role of Current Techniques and Concepts in Peripheral Nerve Repair

**DOI:** 10.1155/2016/4175293

**Published:** 2016-01-20

**Authors:** K. S. Houschyar, A. Momeni, M. N. Pyles, J. Y. Cha, Z. N. Maan, D. Duscher, O. S. Jew, F. Siemers, J. van Schoonhoven

**Affiliations:** ^1^Division of Plastic and Reconstructive Surgery, Department of Surgery, Stanford School of Medicine, Stanford, CA 94305, USA; ^2^Clinic for Plastic and Reconstructive Surgery, Bergmannstrost Halle, 06112 Halle, Germany; ^3^Clinic for Hand Surgery, Rhön-Klinikum AG, 97616 Bad Neustadt an der Saale, Germany; ^4^Orthodontic Department, College of Dentistry, Yonsei University, Seoul, Republic of Korea; ^5^Section of Plastic and Reconstructive Surgery, Department of Surgery, Johannes Kepler University Linz, 4040 Linz, Austria

## Abstract

Patients with peripheral nerve injuries, especially severe injury, often face poor nerve regeneration and incomplete functional recovery, even after surgical nerve repair. This review summarizes treatment options of peripheral nerve injuries with current techniques and concepts and reviews developments in research and clinical application of these therapies.

## 1. Introduction

Despite the progress in understanding the pathophysiology of peripheral nervous system injury and regeneration, as well as advancements in microsurgical techniques, peripheral nerve injuries are still a major challenge for reconstructive surgeons. Injuries of the peripheral nerves are common and debilitating, affecting 2.8% of trauma patients and resulting in considerable long-term disability, especially in hand trauma patients [1]. The occurrence of spontaneous axonal regeneration following an insult reflects the tendency of injured peripheral nerves to recover. While their capacity for regeneration is higher than that of the central nervous system, complete recovery is fairly infrequent, misdirected, or associated with debilitating neuropathic pain [1]. In fact, satisfactory outcomes are usually limited to relatively minor injuries and reflect neurapraxia or axonotmesis. A lacerated nerve has no chance of spontaneous recovery, and the discontinuity must be microsurgically repaired. Even patients undergoing immediate nerve repair are subject to a lengthy denervation period of the distal target, given that the rate of regeneration approaches 1 mm/day in humans [2]. The peripheral nervous system (PNS) is also affected by age-related changes. Structural and biochemical changes that result in a slowly progressive loss of neurons and nerve fibers lead to decreased regenerative and reinnervating capabilities of nerve fibers in aged subjects. Achieving better outcomes depends both on the advancements in microsurgical techniques and on the introduction of molecular biology discoveries into clinical practice. The field of peripheral nerve research is dynamically developing and concentrates on more sophisticated approaches tested at the basic science level. In this chapter we review future directions in peripheral nerve reconstruction focusing on tolerance induction and minimal immunosuppression for nerve allografting, cell based supportive therapies, and bioengineering of nerve conduits.

## 2. Classification of Nerve Injuries

The classification of nerve injuries, originally proposed by Seddon in 1943 (three degrees of injury) and Sunderland in 1951 (five degrees of injury), was subsequently expanded by Mackinnon to include a sixth category representing a mixed injury pattern [3]. The level and degree of injury are important in determining treatment. In the Mackinnon classification, first-, second-, and third-degree injuries have the potential for recovery and for the most part do not require surgical intervention [3]. With a first-degree injury, the nerve temporarily loses conductive signaling activity but the axonal bundle remains intact. This type of injury recovers function within three months. A second-degree injury recovers slowly at a rate of 1 inch per month. With this injury type, the axon suffers damage but the connective tissue surrounding the nerve remains intact. Because of this, the nerve is able to regenerate completely. Third-degree injuries involve injury to the endoneurium while sparing the epineurium and perineurium. These injuries do not recover well without surgical intervention. Recovery is slow and often incomplete. Fourth- and fifth-degree injuries are more severe and will not recover without surgical intervention. In fourth-degree injury, only the epineurium is intact and in fifth-degree injury, the entire nerve is transected. A sixth degree represents a combination of any of the previous five levels of injury.

The classification of injury type is useful to understand the prognosis and the likelihood of complete recovery. Because of the longitudinal nature of crushing injuries, different levels of nerve injury can be seen at various locations along the nerve. This is the most challenging nerve injury for the surgeon as some fascicles will need to be protected and not “downgraded,” whereas others will require surgical reconstruction (Table 1).

## 3. Pathophysiology of Nerve Degeneration and Regeneration

**Figure 1:**
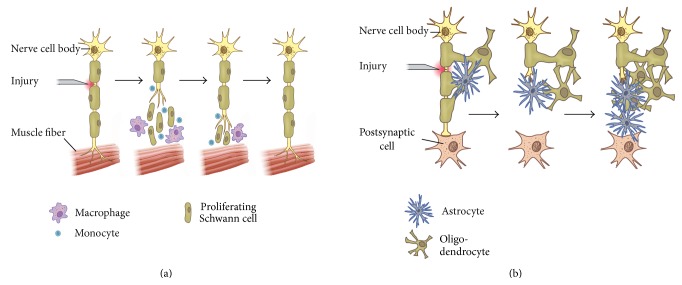
(a) In the PNS, support cells aid neuronal regeneration. Proliferating Schwann cells, macrophages, and monocytes work together to remove myelin debris, release neurotrophins, and lead axons toward their synaptic targets, resulting in restored neuronal function. (b) In the CNS, however, the few neurons that survive axotomy attempt regeneration and subsequently meet an impenetrable glial scar composed myelin and cellular debris, as well as astrocytes, oligodendrocytes, and microglia. Fibroblasts, monocytes, and macrophages may also be present in the glial scar. Consequently, regenerating neurons in the spinal cord are blocked from reaching their synaptic target.

After a nerve is severed, the distal portion begins to degenerate as a result of protease activity and separation from the metabolic resources of the nerve cell bodies. Wallerian degeneration of the distal stump involves invasion by myelomonocytic cells that destroy myelin and initiate mitosis in Schwann cells. Degeneration of the distal axon ends presumably occurs by autolytic mechanisms (Figure 1(a)). The cytoskeleton begins to breakdown, followed by dissolution of the cell membrane. The proximal end of the nerve stump swells but experiences only minimal damage via retrograde degradation [4]. After the cytoskeleton and membrane degrade, Schwann cells surrounding the distal portion of the axon shed their myelin lipids. Phagocytotic cells, such as macrophages and Schwann cells, clear myelin and axonal debris. In addition to clearing myelin debris, macrophages and Schwann cells also produce cytokines (interleukin-6), which enhance axon growth [5]. Following debris clearance, regeneration begins in the proximal severed end and continues toward the distal stump. New axonal sprouts usually emanate from the nodes of Ranvier, which represent nonmyelinated areas of axon located between Schwann cells. The Schwann cells help to guide the cytoplasmic extensions of the axonal sprout between the basement membrane of two nerve ends [6]. Functional reinnervation requires that axons extend until they reach their distal target. In humans, axon regeneration occurs at a rate of ~1 mm/day; thus, significant injuries can take months to heal [7]. This reinnervation is not without complication or resultant dysfunction. Uncontrolled branching of growing axons at the lesion site and misdirection of axons and target organ reinnervation errors are common complications [8]. The central nervous system's regeneration capacity is not very appreciable compared to the peripheral nervous since. Although astrocytes within the CNS proliferate in a similar manner to that of Schwann cells in the PNS, instead they become “reactive astrocytes” in the CNS, producing glial scars that inhibit regeneration (Figure 1(b)).

## 4. Nerve Repair

Direct nerve repair with epineural microsutures is still the gold standard surgical treatment for severe axonotmesis and neurotmesis injuries. Epineural repair is performed when a tension-free coaptation can be achieved in a well-vascularized bed which was developed by Millesi. Gross fascicular matching between the proximal and distal nerve ends results from lining up both the internal nerve fascicles and the surface epineural blood vessel patterns.

Other repairs include grouped fascicular repair requiring intranerve dissection and direct matching and suturing of fascicular groups [9]. This is more practical distally in a major peripheral limb nerve. However, the theoretical advantages of better fascicle alignment with this technique are offset by increased trauma and scarring to the healing nerve internally due to the presence of permanent sutures. Despite its anatomical attractiveness, overall group fascicular repair is no better than epineural repair in functional outcomes [10].

## 5. Surgical Alternative to Nerve Repair: Nerve Transfers

The definition of nerve transfer is the surgical coaptation of a healthy nerve donor to a denervated nerve. This is usually reserved for important motor nerve reconstruction although it can equally be applied to critical sensory nerves. Nerve transfers use an expendable motor donor nerve to a less important limb muscle [11]. The nerve is cut and then joined to the injured distal end of the prioritized motor nerve.

The benefits of nerve transfers are well described. In most cases there is only one neurorrhaphy site; with nerve grafts, there are two. In addition, nerve transfers minimize the distance over which a nerve has to regenerate because it is closer to the target organ and is more specific [12]. Pure motor donors are joined to motor nerves and sensory donors to sensory nerves, optimizing regeneration potential. As opposed to a tendon transfer, when a nerve transfer is successful, recovered function is similar to the original muscle function because synchronous physiologic motion may be achieved. With quicker nerve recovery, more rapid motor reeducation is also possible. The goal is to maximize functional recovery with fast reinnervation of denervated motor targets. The most common applications of motor nerve transfers include the restoration of elbow flexion, shoulder abduction, ulnar-innervated intrinsic hand function, radial nerve function, and smile reconstruction from facial nerve palsy [13]. Aszmann et al. reported about a case series of three patients who were treated successfully with bionic reconstruction to restore hand function after brachial plexus injury [14]. Another publication discussed the role and mechanism of brain plasticity in nerve regeneration [15].

## 6. Role of Alternative Repair Strategies

### 6.1. Nerve Conduits

**Figure 2:**
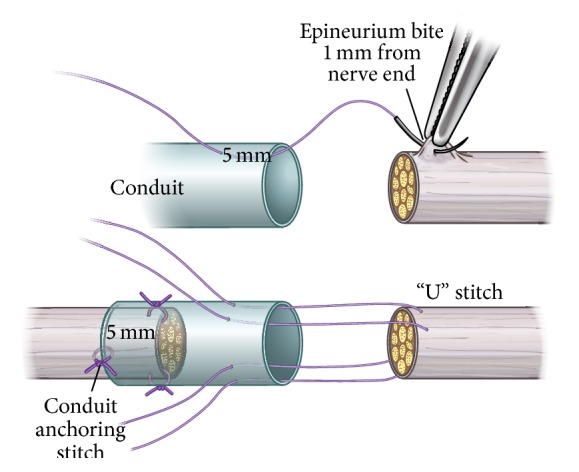
Picture showing a vein conduit used to bridge traumatic nerve laceration.

Studies show that nerves will regenerate across a short nerve gap through various conduits, such as veins, pseudosheaths, and bioabsorbable tubes [16].  Figure 2 demonstrates a commonly available vein conduit used to bridge a nerve laceration. When a hollow nerve conduit is used to repair a severed peripheral nerve, an additional step for regeneration is required [17]. After injury, a fibrin bridge is formed through the conduit and across the defect site. This fibrin cable includes macrophages and other cells thought to be involved in debris clearance. The fibrin bridge retracts as Schwann cells and capillaries begin to grow across the gap, and regeneration proceeds as normal [18]. It is not clear if the formation of a fibrin cable also occurs in the absence of a conduit or when a conduit contains an internal matrix.

The characteristics of the ideal nerve conduit include low antigenicity, availability, and biodegradability. The benefits of vein grafts have been used to reconstruct distal sensory nerve defects of less than 3 cm. Sensory results with vein grafts have been acceptable but not as good as conventional grafting [19]. For this reason, vein grafts are recommended only for reconstruction of noncritical nerve gaps of less than 3 cm [20].

Nerve regeneration across a 3 cm gap through a biodegradable polyglycolic nerve tube has been demonstrated in the primate model and in a clinical trial [9]. Clinical recovery was comparable to that across an autologous nerve graft. The insertion of a short piece of nerve graft material into the center of the conduit will enhance regeneration by providing a local source of trophic factors [21]. The ready availability of biodegradable synthetic grafts to span short nerve gaps would eliminate the morbidity associated with nerve graft harvest and would capitalize on the potential benefits of neurotropism in directing nerve regeneration. Synthetic nerve conduits are now available for reconstruction of small diameter nerves with a gap ≤ 3 cm, or with large diameter nerves with gaps ≤ 0.5 cm [22]. Lohmeyer et al. could show that the long-term recovery of sensibility after digital nerve tubulization depends on the nerve gap length with better results in those <10 mm. Nerve regeneration after tubulization seems not to be terminated after 12 months. Manoli et al. demonstrated that muscle-in-vein conduits may be a good alternative solution to autografts for the reconstruction of digital nerves. Siemers et al. presented various tubulization possibilities, including their limitations. In summary, the use of nerve conduits has evolved from an experimental idea to a clinical reality over the last twenty years.

### 6.2. Nerve Autografts and Allografts

In patients with larger nerve gaps where the injury must be bridged, use of an autograft remains the most reliable repair technique [23]. Whereas nerve conduits rely on fibrin clot stability, a nerve graft provides original internal scaffolding with hundreds to thousands of basal lamina tubes to support Schwann cell and axon migration. The three major types of autografts are cable grafts, trunk grafts, and vascularized nerve grafts [10]. Cable grafts are several sections of small nerve grafts aligned in parallel to connect fascicular groups. Trunk grafts are mixed motor and sensory grafts. Trunk grafts have poor functional results due to their instability and large diameters which inhibits its ability to properly revascularize the center of the graft. Vascularized nerve grafts have the advantage that there is no period of ischemia compared to nonvascularized grafts and the necessity for revascularization is avoided; however there have been conflicting results demonstrating their clinical superiority over nonvascularized grafts. Sensory donor nerves are most often used, with the sural nerve being the most commonly harvested. Furthermore, it is commonly advised to choose a graft that is 10% to 20% longer than the existing nerve gap to ensure a tension-free repair [23]. Although no large clinical studies exist comparing these techniques, in cases where the diameter is mismatched, the most commonly used approach is the use of cable grafts.

Nerve allografts have demonstrated clinical utility in repairing extensive peripheral nerve injuries where there is a paucity of donor nerve material [23]. Allografts used in peripheral nerve injuries are commercially processed to be cell and protein free. This allows the nerve allograft to serve as a scaffold that is repopulated by host axons and Schwann cells over time. As a result, it challenges the immune system for only a limited period of time. Tacrolimus has been successfully used in patients treated with peripheral nerve allografts, with its beneficial effects being explained by its dual function as an immunosuppressive and neuroregenerative agent [24]. Like autografts, the nerve allograft provides a scaffold for nerve regeneration but has the potential for shorter operative time, abundant supply, and lack of donor site morbidity. Potential candidates for peripheral nerve allotransplantation receive nerve allografts from donors that have been screened for ABO blood typing, HIV, and cytomegalovirus [25]. A recent multicenter retrospective study evaluated seventy-six nerve repairs performed at various centers in a relatively heterogeneous group (forty-nine sensory, eighteen mixed, and nine motor) using processed human nerve allograft [26]. Subgroup analysis was performed to determine the influence of nerve type, gap length, patient age, time to repair, age of injury, and mechanism of injury on outcomes. Griffin et al. reported significant recovery in 87.3% of subjects across subgroups using both qualitative and quantitative outcome measures, with no response to treatment in eight of the subjects [27]. There were no graft-related adverse effects. Additionally, the study showed functional recovery in nerve gaps up to 50 mm.

Immunogenicity has historically been a concern with allografts [28]. Although graft Schwann cells display major histocompatibility complexes that incite a T-cell response, host Schwann cell proliferation and irradiation of the graft improve regeneration and histologic outcome in animal models. Karabekmez et al. retrospectively studied short-term sensory recovery after decellularized cadaveric nerve transplantation in seven patients with ten nerve gaps, eight digital and two ulnar sensory [28]. They examined 2-point discrimination and found that all patients recovered 10 mm or better static 2-point discrimination with five good results and five excellent results with no cases of infection or rejection. Although larger randomized studies are needed, for small gaps up to 3 cm, allograft outcomes may be comparable with that of conduits in sensory outcome. Ray et al. reported success in a mouse model with cold preservation for four weeks to decrease immunogenicity [16]. Whereas most studies have focused on sensory recovery, a recent study design compared motor recovery of autograft to allograft and collagen conduit in rat sciatic nerve gap lesions and found autograft superior to allograft at sixteen weeks postoperatively in terms of isometric strength recovery [27]. Allograft and autograft were superior (*p* ≤ 0.05) to collagen conduit. Despite this headway, more development is needed prior to recommending allograft use over autograft for longer nerve gaps. In summary, the current gold standard procedure to bridge damaged peripheral nerves is the use of autologous nerve grafts.

### 6.3. Growth Factors

More recently, studies have demonstrated the efficacy of applying growth factors to the nerve conduit lumen [18]. Studies on the use of various growth factors to promote peripheral nerve regeneration have gradually increased (Table 2), with an improved understanding of neurotrophic components that are released from nerve endings and their effect on nerve growth and differentiation. These neurotrophic factors, expressed at different intervals during nerve regeneration to accelerate axonal growth, include nerve growth factor (NGF), brain derived neurotrophic factor (BDNF), ciliary neurotrophic factor (CNTF), and insulin-like growth factor-1 (IGF-1), all of which are secreted from Schwann cells [29].

Fibroblast growth factors (FGFs) have a significant role in cell growth and regeneration and are released from damaged nerve ending [30]. Subsequent studies have worked on combining FGF with structural components. Midha et al. used synthetic tube bridge material with 10 lg/mL of FGF-1 and collagen matrix in a nerve defect of 10 mm and determined an increase only in regeneration in comparison with the collagen matrix group [31]. After facial nerve decompression surgery, Hato et al. applied basic-FGF-impregnated biodegradable gelatin around the regenerating exposed nerve and found an increased complete recovery rate compared to conventional surgery, demonstrating the efficacy of FGF in enhancing peripheral nerve regeneration [32]. While the mechanism for aFGFs efficacy is unclear, there are various theories, including an increase in the number of Schwann cells in the field of the nerve cut, an enhanced neovascular response, a survival advantage for the injured nerve cells, and a trophic effect for ensuring the continuity of newly occurred axons.

Neuron growth factor (NGF) plays an important role in physiological nerve healing and regeneration [33]. NGF immobilized on gelatin membranes, or PLGL scaffolds, promotes Schwann cell adhesion and survival in vitro and neurite outgrowth from pheochromocytoma cells, indicating this approach is potentially useful for the generation of nerve conduits for clinical nerve repair. Insertion of Schwann cells into the conduit is a relatively simple method that also increases production of NGF [34]. First evidences indicate that a controlled release of NGF by microspheres, or by adenoviruses expressing this factor, increases the functional recovery of injured peripheral nerves. Although the organic solvent used for the NGF-microspheres production might compromise NGF activity, the possibility of directly adding NGF to nerve conduits has not been studied as an alternative for local treatments.

Glial growth factor (GGF), another epidermal growth factor, is released from neurons that has been shown to induce Schwann cell proliferation [35]. It plays a role in the interaction between neuronal and glial cells with respect to peripheral nerve healing. GGF applied into a conduit for defects of 2–4 cm in a rabbit peroneal nerve model increased the number of newly formed Schwann cells, significantly improved axonal regeneration, and considerably decreased the muscle mass lost in comparison with the control group [36]. Ciliary neurotrophic factor (CNTF) is contained in the cytoplasm of myelin Schwann cells and increases neuron survival following axotomy [37]. It is directly released from the circumference of the neuron. It has been used within silicone conduits in rat sciatic nerve defects of 10 mm and has increased the diameter and number of axons, myelinization, and motor nerve conduction rate, thereby increasing the amplitude of muscle action in comparison with controls [37].

Vascular endothelial growth factor (VEGF) is best described for its influence on endothelial cell biology and its role in neovascularization; however, it has been reported that VEGF also has positive effects on nerve regeneration [38]. Hobson et al. demonstrated that a laminin-based gel (Matrigel) and VEGF (500–700 ng/mL) applied to a silicone conduit in a 1 cm rat sciatic nerve defect enhanced blood vessel penetration around nerve cells and increased Schwann cell migration and axonal regeneration [39]. In summary, impregnation of neurotrophic factors such as NGF or FGF-1 into fabricated collagen/laminin fibrils represents an exciting new therapeutic paradigm in combination with current surgical techniques.

### 6.4. Neural Tissue Engineering

Advances in bioengineering provide additional biologically stable materials that have the ability to integrate growth-enhancing agents or factors into the lumen of the conduit. One major drawback for current nerve graft techniques is the requirement of a secondary donor site and subsequently injury and repair site. A combination of tissue engineering with cellular seeding could serve as an alternative for nerve grafts without the need for a secondary surgery. An ideal nerve conduit requires a scaffold that is porous, biocompatible, biodegradable, conductive, and resistant to infections [40]. A major challenge is developing a scaffold that can correctly combine all of the required properties. Additionally, cellular and extracellular matrix alignment is critical for adequate function of biological tissues. Within the nervous system, collagen fibers orientate in response to force vectors and also strengthen the ECM. Much of the research in tissue engineering has focused on the development of anisotropic scaffolds that provide the support associated with properly aligned ECM [41]. With a nerve graft, the aligned Schwann cells are able to support and guide the regenerating neurites at the repair site, and the recreation of this anisotropic 3D cellular architecture is the focus of much research in peripheral nerve repair. Current techniques to develop anisotropic cellular substrates conducive to neural regeneration incorporate the use of Schwann cell-seeded aligned fibers made from synthetic polymers [42], collagen based microstructured 3D nerve guide with longitudinal channels seeded with Schwann cells [43], acellular nerve matrix seeded with adipose-derived stem cells [44], and micropatterned conduits comprised of polylactide tubes seeded with neural stem cells [40].

## 7. Considerations for Optimizing Stem Cell Therapy for Peripheral Nerve Repair

While a variety of strategies have been developed to enhance neuroregeneration in response to trauma, circumstances in which cell loss is extensive, such as following significant injury or in response to degenerative diseases of the nervous system, will likely require complete cell replacement. In the hope of regenerating tissue through cell replacement, many efforts have focused upon the use of stem cells as a source of “replacement” cells [45]. In this case, the stem cells could be harvested before the reconstructive surgery. Neural stem cells have been isolated from rodent brain, spinal cord, skeletal muscle, and bone marrow.

Bone marrow stromal cells, also known as mesenchymal stem cells (MSCs), have been transdifferentiated successfully into neural cells [46]. As MSCs can be isolated relatively easily from bone marrow aspirates and expanded in culture, they provide an interesting alternative to Schwann cell transplantation. Upon implantation of the NC into a rat sciatic nerve gap of 5 mm, functional recovery in terms of conduction velocity and sciatic functional index was significantly improved as compared with MSC-free control NC [47]. Functional recovery was similar to that obtained with a NC loaded with Schwann cells. The similar outcome of the two cell-loaded NC groups is quite remarkable considering that only about 5% of the MSCs transdifferentiated into a Schwann cell-like phenotype, while the major cell population maintained an undifferentiated phenotype, as evidenced by S100 protein staining [47]. The paracrine effects of MSCs likely play a role in the observed phenotype, along with deposition of basal lamina components [48]. Although the mechanism of MSC transdifferentiation and the molecular cross talk between MSCs and peripheral nerves are not fully understood, MSCs may become a promising and abundant therapeutic source for cell based approaches to nerve regeneration [49].

One study compared the neural differentiation capacity between human muscle-derived stem cells and human adipose-derived stem cells (hADSCs) in vitro and found that neural differentiated hADSCs had significantly higher levels of mRNA and protein of neuronal marker *β*-tubulin III and glial marker GFAP compared to neural differentiated hMDSCs demonstrating that hADSCs have a higher differentiation capacity compared to hMDSCs [50]. In murine models, human muscle-derived stem cells have also shown the potentiation to adopt into neuronal tissues [51]. When adult human skeletal muscle-derived stem cells (hMDSCs) were transplanted into a sciatic nerve injury site, engraftment of hMDSCs promoted axonal regeneration which led to functional recovery without any adverse effects 18 months after the transplant [51]. These data demonstrate the potential to use hMDSCs in the treatment of human neuropathies.

Incidentally, stem cells have also been isolated from hair follicles and have adopted Schwann cell characteristics when placed between the stumps of a transected peripheral nerve [52]. However, extraction of a high number of hair follicle stem cells seems more laborious than harvesting MSC. Interestingly, 2–5 weeks after transplantation, stem cells implanted in injured rat spinal cords have survived; differentiated into neurons, astrocytes, and oligodendrocytes; and migrated up to 8 mm from the lesion. Rats receiving the transplanted stem cells showed improved functional recovery [53]. Similarly, other studies have also found that stem cells implanted into injured spinal cord differentiate into neurons and glial cells [4]. Consequently, it has been suggested that the environment is a greater factor in neural stem cell fate than the intrinsic properties of the cell. Greater control over stem cell differentiation, by in vitro treatments or by using stem cells that are restricted to the neuronal lineage, may allow stem cell transplantation to yield more predictable results. In summary, bone marrow stem cells have been shown to be capable of differentiating into neuronal and glial phenotypes and the clinical use of bone marrow stem cells should be investigated in the future.

## 8. Electrical Stimulation

There have been limited reports of applying electrical fields/gradients across a repaired peripheral nerve to speed up axonal regeneration. Animal studies demonstrate that as little as one hour of direct nerve electrical stimulation immediately after repair of a transected femoral nerve in the rat promotes a dramatic increase in the kinetics of target muscle reinnervation [54].

In a clinical pilot study, one hour of electrical stimulation was applied after median nerve decompression at the wrist for 21 patients with carpal tunnel syndrome and thenar atrophy [55]. The electrical stimulation group showed evidence of accelerated axonal regeneration and reinnervation evidenced by motor unit number estimation and sensory and motor nerve conduction studies.

## 9. Conclusions

The requirements for functional nerve regeneration are complex. However, through the combined efforts of scientists and engineers from a variety of disciplines, experimental work in this field has made great progress. While nerve grafting is often the clinical gold standard for larger nerve injuries, recent developments utilizing growth factors, stem cells, and nerve conduits should extend the realm of possibilities of peripheral nerve repair. New potential targets for novel therapies have been discovered through an increased understanding of the molecular biology of neural development and regeneration. Tissue engineering and nanotechnology are suggesting new research therapeutic approaches, potentially orientated to accelerate nerve regeneration and recovery of nerve functionality. As discussed in this review, many significant advances in nerve repair and regeneration have been achieved. Further studies will continue to advance the field of therapeutics in regeneration of the PNS. We are on the verge of a breakthrough in our current understanding that can potentially transform the field of peripheral nerve repair, ultimately offering new options to patients with severe nerve injuries.

## Figures and Tables

**Table 1:** Neurosensory impairment classification according to Sunderland and Seddon.

Classification of nerve injury				
Sunderland	Seddon	Injury	Neurosensory impairment	Recovery potential
I	Neuropraxia	Intrafascicular oedema, conduction block	Neuritis, paresthesia	Full (1 day to 1 week)
		Possible segmental demyelination	Neuritis, paresthesia	Full (1 to 2 months)

II		Axon severed, endoneurial tube intact	Paresthesia, episodic dysesthesia	Full (2 to 4 months)

III	Axonotmesis	Endoneurial tube torn	Paresthesia, dysesthesia	Slow, incomplete (12 months)

IV		Only epineurium intact	Hypoesthesia, dysesthesia, and neuroma formation	Neuroma in continuity

V	Neurotmesis	Loss of continuity	Anaesthetic, intractable pain, and neuroma formation	None

VI		Combination of above	Combination of above	Unpredictable

**Table 2:** Various growth factors to promote peripheral nerve regeneration.

Growth factor	Main target
NGF	Sensory neurons and small axons
BDNF	Sensory neurons and large axons
CNTF	Sciatic nerve
IGF-1	Inflammatory cells and sensory and motor neurons
VEGF	Vascular endothelial cells
